# Extracellular Vesicles as Biomarkers in Liver Disease

**DOI:** 10.3390/ijms232416217

**Published:** 2022-12-19

**Authors:** Rocío Muñoz-Hernández, Ángela Rojas, Sheila Gato, Javier Gallego, Antonio Gil-Gómez, María José Castro, Javier Ampuero, Manuel Romero-Gómez

**Affiliations:** 1 SeLiver Group, Institute of Biomedicine of Seville (IBiS), Virgen del Rocio University Hospital/CSIC/University of Seville, 41013 Seville, Spain; 2 CIBERehd, Instituto de Salud Carlos III, 28029 Madrid, Spain; 3 Servicio de Citometría y Separación Celular, Instituto de Biomedicina de Sevilla Virgen del Rocio University Hospital/CSIC/University of Seville, 41013 Seville, Spain; 4 UCM Digestive Diseases, Virgen del Rocío University Hospital, 41013 Seville, Spain

**Keywords:** extracellular vesicles, biomarkers, liver disease

## Abstract

Extracellular vesicles (EVs) are membrane-derived vesicles released by a variety of cell types, including hepatocytes, hepatic stellate cells, and immune cells in normal and pathological conditions. Depending on their biogenesis, there is a complex repertoire of EVs that differ in size and origin. EVs can carry lipids, proteins, coding and non-coding RNAs, and mitochondrial DNA causing alterations to the recipient cells, functioning as intercellular mediators of cell–cell communication (auto-, para-, juxta-, or even endocrine). Nevertheless, many questions remain unanswered in relation to the function of EVs under physiological and pathological conditions. The development and optimization of methods for EV isolation are crucial for characterizing their biological functions, as well as their potential as a treatment option in the clinic. In this manuscript, we will comprehensively review the results from different studies that investigated the role of hepatic EVs during liver diseases, including non-alcoholic fatty liver disease, non-alcoholic steatohepatitis, alcoholic liver disease, fibrosis, and hepatocellular carcinoma. In general, the identification of patients with early-stage liver disease leads to better therapeutic interventions and optimal management. Although more light needs to be shed on the mechanisms of EVs, their use for early diagnosis, follow-up, and prognosis has come into the focus of research as a high-potential source of ‘liquid biopsies’, since they can be found in almost all biological fluids. The use of EVs as new targets or nanovectors in drug delivery systems for liver disease therapy is also summarized.

## 1. Introduction

More than eight hundred million people suffer from chronic liver disease, which accounts for approximately two million deaths per year worldwide [1], with cirrhosis, viral hepatitis, and hepatocellular carcinoma (HCC) being the leading causes of liver-related deaths. Liver biopsy remains the gold standard diagnostic tool to assess the stage of liver diseases despite its significant disadvantages (low acceptance, highly invasive, and heterogeneous). The lack of non-invasive tools constitutes a significant barrier to the clinical management of liver diseases. 

Circulating extracellular vesicles (EVs), a heterogeneous population of small membrane-encapsulated particles identified in several body fluids such as blood, saliva, and urine, have been proposed as markers for liquid biopsies in several diseases [2,3]. In the past decade, several shreds of evidence have suggested that EVs have a key role in liver disease, since they have been recognized as potent vehicles of intercellular communication due to their capacity to transfer proteins, lipids, and nucleic acids, thereby influencing various physiological functions of the recipient cells [4,5]. In this way, they may contribute to the pathogenesis, initiation, and progression of different liver diseases [6], emerging as a key player in cell–cell communication during acute and chronic liver disease [7]. Previous studies report changes in the number, surface markers, and cargos in the circulating EVs after liver injury, thus representing a potential biomarker for liver disorders [8]. Interestingly, changes in EVs have been demonstrated before histological signs appear, providing strong evidence of their utility as non-invasive tools even for early diagnosis [9].

However, much remains unknown regarding their origin, biogenesis, or secretion. Moreover, different technologies and methodologies have given rise to inconsistencies in the quantification and isolation, making it challenging to compare and extrapolate previous results, which still limit their translation. This review provides critical and up-to-date information on the current knowledge about the use of EVs as biomarkers in liver disease development, its progression, and response to treatment, as well as its use as a potential therapeutic tool.

## 2. Biogenesis, Definition, and Classification of Extracellular Vesicles

**Figure 1:**
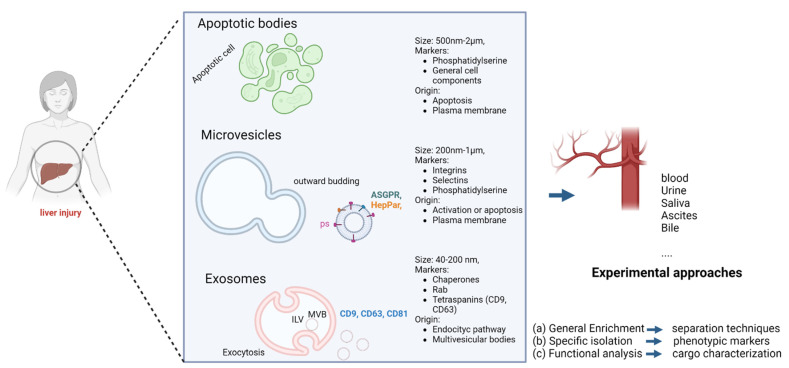
Extracellular vesicle types, biogenesis, and characteristics. (Created with BioRender).

Extracellular vesicles are small vesicles coated with a lipid bilayer membrane released from virtually all cell types under physiological and pathological conditions [4,10]. They contain various surface markers and cargos derived from the parental cells. EVs are classified into three main groups based on their size and biogenesis: exosomes, microvesicles (MVs), and apoptotic bodies (Figure 1) [2]. Therefore, the term “extracellular vesicle” is generally used to cover the three groups mentioned above. 

Exosomes, the EV population most widely studied, ranging from 40–200 nm, are formed by endosomal pathways [11]. Inward protrusions of early endosomes create intraluminal vesicles leading to the formation of multivesicular bodies (MVBs). MVBs fuse to the plasma membrane resulting in the extracellular release of intraluminal vesicles as exosomes [12].

MVs range from 200–1000 nm and are generated in response to stimulation via direct exocytosis [13]. MV release is initiated by an increase in intracellular Ca^2+^ concentration, which leads to the activation of scramblase and calpain, leading to a loss of membrane phospholipid asymmetry and the reorganization of the cytoskeleton. The release of MVs depends on the ATP-mediated activation of P2X7 receptors, which leads to rearrangements of the cell membrane. Specifically, phosphatidylserine (PS), which is usually located/assembled in the inner layer, is externalized via the activation of flippases, generating the release of MVs [14,15]. Based on their biogenesis, they can express different markers that can be detected and used for their identification. Annexin V is widely used for MVs (pan-marker) because of its affinity to PS, and tetraspanins (CD63, CD81, CD9) for exosomes [8].

Cells can secrete EVs through different mechanisms in response to different stimuli. In that sense, their surface markers and cargos may not only reflect their cellular origin, type of damage (i.e., apoptosis, activation…), and mechanism of secretion, but also the pathological state of the parental cell [16,17]. Unlike exosomes and MVs, apoptotic bodies (500–2000 nm) are not related to intercellular communication [18,19] and are not under the focus of this research. A recent consensus stated that EVs may be classified as small (<200 nm) or medium/large vesicles (>200 nm) [19].

## 3. Technology for Characterization and Isolation

There is no clear consensus with regards to the optimal process for quantification, isolation and characterization, thus limiting the translational value of EVs. Related to this, the International Society of Extracellular Vesicles (ISEV) developed guidelines [19] and recommendations in order to support the credibility of EV research by introducing robust reproducibility between studies. Procedures for isolation and characterization include a variety of methods based on physical (size, density, morphology, etc.) or biological properties (cargo, antigen expression). Some of these techniques select the entire EV population independently of cell origin but others can specifically detect EVs derived from specific cell types, and thus related to a specific disease. Each of these methods has its own limitations that must be taken into account and will be briefly described in this section.

(a) Ultracentrifugation is currently the most commonly used technique for EV isolation [20]. Separation is achieved by differences in density and size, using consecutive centrifugations to reduce the number of other particles and concentrate the EVs, so the main advantage is its low cost. However, the type of rotor used heavily impacts the efficiency of the technique, damaging causing the loss of EVs, leading to low reproducibility [21]. (b) Nanomembrane ultracentrifugation spin devices have recently been used for EV isolation from urine [22] and plasma samples [23] with great success. Despite obtaining a final product with high purity from a low volume of sample, authors have described sample loss during fractionation and selection of the filter [24]. (c) Microfluidics is based on physical and biochemical properties (size, density, immuno-affinity, electrophoretic), allowing for the quantification of EVs and the analysis of surface markers and cargo proteins. (d) Immunobeads for tissue-specific EV isolation are used to selectively isolate EVs according to their surface markers, but prior knowledge of the EVs’ characteristics is required [25]. Indeed, previous studies reported that beads-based detection is less sensitive in detecting larger EVs and more sensitive for exosomes than flow cytometry [26].

Following EV enrichment, the EVs must be characterized and counted. Nano-tracking analysis (NTA) is widely used to analyze size and concentration but several small particles can contaminate samples and interfere with the quantification [27]. Moreover, its high cost limits its use. Dynamic light scattering (DLS), another technique that depends on the scattering of a laser beam, allows the measuring of particles ranging from 1 nm to 6 nm; however, the profiling of particle sizes is strongly influenced by larger particles [28]. Unlike DLS and NTA, electron microscopy is not appropriate for quantitative analysis but is useful for obtaining high-resolution images to analyze morphology and size. Moreover, the use of gold nanoparticles (GNPs) gives us the possibility to characterize their phenotype [29]. Its disadvantage is it must be stressed that samples have to be fixed and dehydrated before the measurement. Flow cytometry is widely used for characterization, quantification and isolation; different vesicle populations can be analyzed according to the levels of several antigens. Its limitations are small-sized EVs cannot be detected and samples need to be processed before staining (ultracentrifugation, precipitation, magnetic purification, etc.), and the media could be contaminated by microparticle-like debris. Despite all of that, flow cytometry is considered the most promising technique in meeting the criteria for EV quantification and phenotype characterization (using cell-specific antibodies).

## 4. Extracellular Vesicles as a Biomarker in Liver Disease

### 4.1. Non-Alcoholic Fatty Liver Disease (NAFLD)

NAFLD is the most common chronic liver disease worldwide, affecting about a quarter of the world’s adult population [30]. The diagnosis of steatohepatitis (NASH), a more severe form of the disease, requires a histological examination to confirm the presence of ballooning and inflammation; thus, the development of non-invasive biomarkers in NAFLD has generated considerable attention in the scientific community. Previous studies reported the role of EVs in NAFLD development, such as hepatocyte-derived sphingosine 1-phosphate (S1P)-containing EVs with pro-inflammatory activity in NASH [31]. Indeed, hepatocyte-derived EVs released from damaged hepatocytes in experimental models of NASH activate non-parenchymal cells, such as endothelial, stellate, and hepatic macrophages, contributing to the progression of liver disease [32]. A few studies support the use of several types of EVs for diagnostic purposes in patients with NASH (Table 1). Circulating CD14+ MVs in NAFLD correlated with alanine aminotransferase (ALT) levels and NAScore [6]. Recently, Povero et al. [33] analyzed hepatocyte-derived EVs from pre-cirrhotic and cirrhotic NASH patients, using asialoglycoprotein receptor 1 (ASGPR1) and long-chain fatty acid transport protein 5 (FATP-5) as hepatocyte markers. Levels of ASGPR1 EVs were found to be increased twofold in pre-cirrhotic NASH and threefold in cirrhotic NASH, compared to healthy controls. Furthermore, ASGPR1+ EVs were also found to correlate with the hepatic vein portal gradient (HVPG), being a potential non-invasive biomarker of portal hypertension in patients with NASH and cirrhosis. Finally, a proteomic analysis of circulating EV cargo revealed two feature pairs (IL13Ra1 > TNR4; WISP-1 > BMP-14) with strong predictive power for liver fibrosis and cirrhosis (80% sensitivity and 80% specificity). Platelet-derived MVs have also been proposed for NASH diagnosis, with levels of CD61+ MVs correlating with fat fraction, ballooning, and fibrosis stage in NAFLD patients [34]. Another study used ASGR2 (asialoglycoprotein receptor 2) and CYP2E1 (cytochrome P450 2E1) antibodies as markers of hepatocyte-derived EVs and observed a decrease in ASGR2+ and CYP2E1+ MVs levels after bariatric surgery in 28 biopsy-proven NAFLD patients [8].

### 4.2. Alcoholic Hepatitis

Uncontrolled alcohol consumption results in a liver injury characterized by steatosis, inflammation, hepatitis, and cirrhosis [35]. Alcohol exposure increases the number of circulating EVs of hepatic origin (mostly hepatocytes and hepatic stellate cells (HSCs)). EVs act on target cells (macrophages, endothelial cells, and HSCs) promoting inflammation and fibrosis [3]. Its release is related to a partial inhibition of autophagy promoted by a decreased level of lysosomal-associated membrane proteins LAMP1 and LAMP2 through miR-155 expression [36]. In that sense, it has been demonstrated that alcohol-treated hepatocytes cross-talk with immune cells via microRNAs (miRNAs) contained in exosomes. In alcoholic liver disease (ALD), serum/plasma miR-122 and miR-155 levels are increased and predominantly associated with exosomes [37]. Thus, hepatocyte-derived exosomes with miR-122 increase the secretion of pro-inflammatory cytokines in monocytes [38]. Also, in hepatocytes, alcohol increases the delivery and formation of exosomes containing mitochondrial double-stranded RNA (mtdsRNA) that participate in the production of pro-inflammatory cytokines (IL1B) in Kupffer cells [39]. Related to this, hepatic EVs derived from alcoholic hepatitis mice are able to activate primary HSCs, inducing a-SMA and collagen through upregulating miRNAs and increasing IL1B and IL17 production in a TLR9-dependent manner in macrophages [40]. In this regard, CD40 ligand (CD40L) in EVs, in a caspase-dependent manner in response to alcohol exposure, has a critical role as a mediator of macrophage activation [41]. Furthermore, alcohol-exposed monocytes can communicate with naive monocytes via miR-27a-loaded EVs that program naive monocytes into M2 macrophages [42]. Also, protein cargo in EVs can be important, since, in animal models of alcoholic liver disease, macrophage activation was induced by hepatocyte-derived EVs harboring heat shock HSP90 protein [43]. 

Alcohol has been implicated in fibrosis through the release of a major fibrogenic cytokine, transforming growth factor-beta-1 (TGF-β1), and HSCs activation [44]. A study associated alcohol with the increase of profibrogenic factors through the levels of miR-19b in HSCs and derived exosomes. Interestingly, decreased miR-19b levels in activated HSCs resulted in a change in the expression of other miRNAs (miR-17–92 cluster). However, miR-19b was induced at the plasma and exosomal levels in this alcohol-induced hepatic fibrogenesis model [45]. In addition, elevated levels of EVs have been observed containing CYP2E1 derived from the liver in patients with alcoholism and in alcohol-exposed animals. CYP2E1 activity is associated with oxidative and endoplasmic reticulum stress after alcohol consumption, leading to the activation of apoptotic pathways and toxicity to monocytes and hepatocytes. Thus, these EVs with CYP2E1 cargo could act as a biomarker for liver damage from long-term alcohol exposure [46].

Moreover, EVs secreted from other organs such as intestinal epithelial cells have effects on hepatocytes during acute alcohol injury, highlighting the importance of the gut-liver axis in ALD progression [47].

Lastly, some studies have tried to find soluble markers to diagnose alcoholic hepatitis in a non-invasive manner. The plasma levels and EVs of cytokeratin-18 fragments (M30 and M65) are reliable non-invasive markers of alcoholic hepatitis [48] High levels of CD34+ and ASGPR1+ EVs can be used as markers of non-response to corticosteroid therapy in severe alcoholic hepatitis [49]. Recently, plasma EV concentration and sphingolipid cargo were found to correlate with the severity and mortality of alcoholic steatohepatitis [50].

### 4.3. Viral Hepatitis

EVs are potent modulators of the immune response. In vitro studies showed that hepatocytes infected with replicating HBV release EVs that induced a programmed cell death 1 ligand 1 (PD-L1) expression in monocytes, possibly suppressing host antiviral activity [51]. Notably, Montaldo et al. analyzed EVs in the plasma of HCV patients after direct-acting antiviral therapy, finding that miR204-5p, miR181a-5p, miR143-3p, and miR-122-p were decreased in the EVs from HCV patients compared to healthy donors. After that, EV cargo was determined after 6 months of therapy, and miR204-p and miR143-3p were still different between healthy and HCV-treated patients, indicating that EV-mediated signals could play a causal role in fibrosis progression despite viral eradication [52]. Another study showed that patients with active hepatitis C (ALT > 100 IU/mL) had an elevated number of T cell-derived MPs compared to patients with mild hepatitis C (ALT< 40U/mL) and healthy controls [53]. Our group recently reported a decrease in endothelial and platelet apoptotic MV levels after a sustained virological response in HCV patients, concluding that this may be directly involved in the improvement of inflammation and endothelial dysfunction observed in these patients after HCV eradication [54].

### 4.4. Fibrosis

Besides amplifying inflammation and modulating injury, EVs have also been demonstrated to promote liver fibrosis in NAFLD and ALD [55]. HSCs regulate the establishment and sustaining of liver fibrosis [56] partly due to their ability to chronically secrete EVs. Previous studies indicate that lipotoxic hepatocyte-derived pro-inflammatory miRNA-rich EVs could activate TLR-3 in HSCs [57], inducing their activation and migration [58]. These activated HSCs (aHSCs) in turn release EVs that contain various profibrotic proteins, lipids, and nucleic acids [59]. Moreover, the release of these EVs increases in response to liver injury [60]. Unlike aHSCs, EVs secreted by quiescent HSCs display antifibrotic properties since their cargo is shown to suppress HSC activation. Furthermore, they reduce inflammation, promote cell viability, inhibit hepatocyte apoptosis, and decrease liver transaminase levels, indicating their therapeutic potential [61,62]. Also, in the progression of NAFLD, exosomes from visceral adipose tissue (VAT) were related to fibrosis through TGF-B dysregulation in the hepatocytes and HSCs [63].

Although HCV does not replicate in HSCs, EVs from HCV-infected hepatocytes induce the expression of profibrogenic genes. miR-19a in these hepatocyte-derived HCV-EVs was able to promote fibrosis by targeting SOCS3 which caused the activation of the STAT3–TGF-B signaling pathway [64]. Previous studies aimed at determining the role of platelet and monocyte-derived MVs as biomarkers of fibrosis in biopsy-proven NAFLD patients [65]. CD14+ CD16+ EVs improved the ability of liver fibrosis scores to identify patients with F3/F4 fibrosis in a small preliminary cohort. Weil et al. reported 2.5-fold higher levels of platelet-derived MVs in 10 healthy subjects compared with 90 cirrhotic patients [66]. Finally, another study found that hepatocyte-derived MVs were 4.0-fold and 2.2-fold higher in patients with Child–Pugh C compare with those with Child–Pugh A or B respectively. Indeed, hepatocyte-derived MVs correlated with HVPG and were able to predict 6-month mortality independently of the Child–Pugh score or Model for End-Stage Liver Disease (MELD) [67].

### 4.5. Hepatobiliary Tumors: HCC and CCA

HCC is the most common primary liver cancer, being the fourth cause of cancer-related deaths worldwide [68]. HCC has a poor prognosis due to the lack of early symptoms and the low sensitivity and specificity of available diagnostic tools. Early detection is essential to improving surveillance and the adoption of curative surgical therapies. In the same scenario, the earlier the cholangiocarcinoma (CCA) detection, the more opportunities there are for curative treatments. In the context of cancer, the role of EVs has emerged as another promising strategy for liver cancer surveillance. Besides cell-to-cell contact, intercellular communication also happens through EVs to set up and modify tumor microenvironments. EVs are released by cancer cells in order to promote tumor growth and improve the tumor microenvironment for the spreading of these cells [69]. EVs are present in circulation at the early and advanced stages of the disease. The stability and integrity of EVs and their molecular cargos may serve as useful early-stage cancer diagnostic biomarkers and therapeutic approaches [70,71,72].

Current guidelines suggest the need for non-invasive tools for the diagnosis of HCC and CCA [73]. Circulating MV levels were found to increase in HCC patients in comparison to cirrhotic patients. Furthermore, they correlated with HCC tumor size, pathological type, and TNM stages, tending to a decrease after surgical intervention [74]. Several HCC-associated surface markers have been used to isolate and quantify liver tumor EVs. As depicted in Table 1, Julich-Haertel and colleagues showed that the combination of annexin V+ EpCAM+ ASGPR1+ CD133+ taMPs allowed one to distinguish liver malignancies and cirrhosis. Furthermore, EpCAM+ ASGPR1+ and annexin V+ were increased in liver cancer (HCC and CCA) compared to cirrhotic patients. In addition, 7 days after tumor resection, EpCAM+ ASGPR1+ annexin V+ levels significantly decreased, showing a strong association with tumors [75]. A high expression of MMP-7-EVs could be a marker for the differential diagnosis of CCA [76]. Regarding early diagnosis, three EV subpopulations, EpCAM+ CD63+, CD147+ CD63+, and GPC3+ CD63+ were highly associated with the early diagnosis of HCC (AUROC of 0.95 (95% CI = 0.90–0.99) with a sensitivity of 91% and a specificity of 90%) [77]. Another pan-cancer marker was proposed for the diagnosis of HCC and CCA, such as EpCAM+ CD147+ EVs which were increased in HCC, CCA, and other cancers [75]. A complex technique integrating covalent chemistry-mediated EV capture/release, multimarker antibody cocktails, nanostructured substrates, and microfluidic chaotic mixers showed that purified EpCAM, ASGPR1, and CD147 EVs have a 10-gene HCC-specific signature that allows one to distinguish HCC patients from at-risk cirrhotic patients (AUROC: 0.93 (95% CI, 0.86–1.00; S: 94.4% and S: 88.5%)) [78]. Early recurrence after liver resection was related to higher levels of Hepar-1+ microparticles before surgery, suggesting its potential role as a prognostic biomarker [79]. An emerging body of evidence supports the idea that platelets have an important role in carcinogenesis, mainly in HCC development [80,81]. In fact, platelet-derived EVs have also been related to colon cancer [82], but further studies are needed in liver tumors.

The content of cancer-derived EVs significantly differs from that of healthy cells, including different types of RNA such as miRNA, lncRNA, and cancer-specific proteins [73]. miR-122 EVs allowed the differentiation of HCC from liver cirrhosis (AUC:0.990, 95% CI, 0.945–1.00). In addition, the combination of miRNA-122, miRNA-148a, and Alpha-fetoprotein (AFP) increases diagnostic accuracy (AUC:0.931, 95% CI, 0.857–0.973), suggesting that the serum vesicle microRNA signature alone or in combination with available markers could be used as a screening tool for HCC [83]. Higher miR-21 in the circulating EVs of HCC patients are better markers than serum miR-21 in differentiating HCC from cirrhotic and healthy patients [84,85]. Another cluster of miRNAs, miR-18a, miR-221, miR-222, miR-224, miR-101, miR-106b, miR-122, and miR-195, were found to have an increase in exosomes from HCC [73]. Inside purified ASGPR1+ EVs, four miRNAs, miR-10b-5p, miR-21-5p, miR-221-3p, and miR-223-3p were found to be increased in those patients with lower AFP levels [86]. The lncRNA LINC00853 in EVs showed a good diagnostic capacity for HCC (AUC:0.934, 95% IC 0.887–0.966) [87]. Another study found that lnc85 was higher in the exosomes of HCC patients with high and low levels of AFP compared to the healthy control and liver cirrhosis (AUC:0.869) [88]. Recently, a study showed that cancer-associated fibroblast (CAF)-derived MVs can be implicated in HCC progression. The survival rate in patients with low antitumoral miR-150-3p levels in plasma CAF-derived exosomes was significantly poor compared to patients with high miR-150-3p levels. [89]

Regarding CCA, it is well known that bile EVs were significantly higher in CCA patients [90]. A proteomic study showed that EVs from CCA patients expressed a specific protein profile showing potential usefulness as a diagnostic tool [91]. Many studies have shown that EVs are involved in the development and progression of liver cancer. Further pieces of evidence to better understand the role of EVs in diagnosis and prognosis are needed.

**Table 1:** Clinical studies on EVs as biomarkers in patients with liver disease.

Liver Disease	Surface Marker and/or Cargo	Sample Size	OUTCOMES	Methods	Ref.
NAFLD	CD14+	NAFLD (n = 67); control (n = 44)	Patients with NAFL or NASH had significantly higher levels of CD14+ MVs (CD14+), which mediate the pathogenesis of NASH.	Flow cytometry	Kornek M. et al. Gastroenterology 2012. [6]
NAFLD	ASGR2 or CYP2E1	NAFLD patients pre- and post-weight loss (n = 22); control (n = 6)	Plasma levels of EVs and hepatocyte-derived EVs are dynamic and decrease following NAFLD resolution due to weight loss surgery.	Nanoparticle tracking analysis	Nakao Y et al. Nanomedicine 2021 [8]
NASH with and without fibrosis	SLC27A5 ASGPR1	Pre-cirrhotic NASH (n = 25); cirrhotic NASH (n = 25); control (n = 25)	Levels of ASGPR+ EVs were found to be increased 2-fold in pre-cirrhotic NASH and 3-fold in cirrhotic NASH compared to healthy controls.	Differential centrifugation, size exclusion; Chromatography and flow cytometry	Povero D et al. Hepatol Commun. 2022. [33]
Alcoholic hepatitis	miR-155	Cirrhosis (n = 6); control (n = 5)	miR-155 as a mediator of alcohol-related regulation of autophagy and exosome production in hepatocytes and macrophages.	ExoQuick and nanoparticle tracking analysis	Babuta M at al. Hepatology 2019. [36]
Alcoholic hepatitis	miR-122	ALD (n = 11)	Exosomes isolated from sera after alcohol consumption or from in vitro ethanol-treated hepatocytes contained miRNA-122.	Nanoparticle tracking analysis	Momen-Heravi F et al. Sci Rep. 2015. [38]
Alcoholic hepatitis	CYP2E1	ALD (n = 14); control (n = 9)	Alcohol (ethanol) and/or its metabolites increased the amounts of EV proteins, including CYP2E1 and other P450 isoforms, that were secreted possibly from damaged hepatocytes.	Ultracentrifugation and ExoQuick	Cho YE et al. Hepatol Commun. 2017. [46]
Alcoholic hepatitis	CD3 CD4, CD68 CD11b, CD45 CD34, and ASGPR.	ALD (n = 101), 71 responders and 30 non-responders; control (n = 20)	Pre-therapy levels of CD34+ and ASGPR+ microvesicles are reliable non-invasive markers of steroid nonresponse and mortality in patients with severe alcoholic hepatitis.	Flow cytometry	Sukriti S et al. Aliment Pharmacol Ther. 2018. [49]
Alcoholic hepatitis	miR-155	ALD (n = 8); control (n = 6)	The alcohol-related increase in number of circulating exosomes was observed in sera of human AH patients.	NanoSight and western blotting	Sehrawat TS, et al. Hepatology. 2021. [50]
Viral hepatitis	CD9, CD63, CD81/miR204-5p, miR181a-5p, miR143-3p, miR93-5p, miR122-5p	HCV (n = 16), before (T0) and after treatment (T6); control (n = 15)	Antifibrogenic miR204-5p, miR181a-5p, miR143-3p, miR93-5p, and miR122-5p were statistically underrepresented in T0 EVs compared to HD EVs, while miR204-5p and miR143-3p were statistically underrepresented in T6 EVs compared to control EVs.	Microbeads, proteomic, and western blot.	Montaldo C, et al. J Hepatol. 2021. [52]
Viral hepatitis	CD11a, CD14, CD147, and annexin V	Active hepatitis (n = 12); mild hepatitis (n = 10); and control (n = 8)	Patients with active hepatitis C had a significant increase in circulating MPs derived from CD4+ as well as CD8+ T cells compared to patients with mild hepatitis C and healthy controls, respectively.	Flow cytometry	Kornek et al. Hepatol. 2011. [53]
Viral hepatitis	CD31, CD41, and annexin V	HCV (n= 114)	Levels of both EMPs and PMPs decreased after sustained virological response and at follow-up.	Flow cytometry	Muñoz-Hernández R et al. Clin Transl Gastroenterol. 2020. [54]
Fibrosis	CD41a, CD42b, CD31, CD105, CD14, CD16, and CD284	NAFLD with liver fibrosis (n = 26)	CD14+ and CD16+ EVs show potential capacity to predict liver fibrosis severity.	Flow cytometry	Welsh JA, et al. J Leukoc Biol 2018. [65]
Cirrhosis	CD31, CD41, CD235a+, and annexin V	Noninfected cirrhotic patients (n = 90); control (n = 10)	Microvesicle levels, mostly platelet-derived, were 2.5-fold higher in healthy volunteers compared with cirrhotic patients. Circulating small AV platelet-derived MV levels were lower in cirrhotic patients and inversely correlated with MELD score.	Flow cytometry	Weil D, et al. Clin Transl Gastroenterol. 2021 [66]
Cirrhosis	CD31, CD41, CD62, CD144, cytokeratin-18, and annexin V	Cirrhotic patients (n = 139)	Hepatocyte MV levels were 4.0-fold and 2.2-fold higher in patients with Child–Pugh C compared to those with Child–Pugh A or B liver disease, respectively.Hepatocyte MV levels correlated with HVPG but cannot identify patients with HVPG > 10 mmHG. Hepatocyte MV level > 65 U/L predicted 6-month mortality independently of Child–Pugh score and MELD score.	Flow cytometry and Elisa	Payancé A, et al. Hepatol. 2018. [67]
Hepatobiliary Tumors (HCC)	-	HCC patients (n = 55); cirrhosis (n = 40); and controls (n = 21)	MV levels were significantly reduced in the 1-month post-operative samples compared to those in the pre-operative samples. MV levels showed better performance than AFP for early detection of HCC.	Bicinchoninic acid assay	Wang W, et al. Cancer Biomark. 2013. [74]
Hepatobiliary Tumors (HCC and CCA)	EpCAM, CD147, ASGPR, CD133, and annexin V	Liver cancer (n = 172); cirrhosis (n = 54); and control (n = 202)	Annexin V+ EpCAM+ CD147+ taMPs were elevated in liver cancer (HCC and CCA). Annexin V+ EpCAM+ ASGPR1+ taMPs were increased in liver cancer compared to patients with cirrhosis. Annexin V+ EpCAM+ ASGPR1+ CD133+ taMPs allowed the distinction of liver malignancies.	Flow cytometry	Julich-Haertel H, et al. J Hepatol. 2017. [75]
Hepatobiliary Tumors (HCC)	EpCAM, CD63, CD147, GPC3, ASGPR 1	Training cohort (n = 106) and validation cohort (n = 72)	EpCAM+ CD63+, CD147+ CD63+, and GPC3+ CD63+ were highly associated with early diagnosis of HCC (AUROC of 0.95 (95% CI = 0.90–0.99) with a sensitivity of 91% and a specificity of 90%).	Flow cytometry	Sun N, et al. Carcinoma. H Hepatol. 2022. [77]
Hepatobiliary Tumors (HCC)	PKH26	HCC patients (n = 36); cirrhosis cohort (n = 26); NASH (n = 26); healthy donors (n = 38), (n = 23); HBV/HCV without liver cirrhosis (n = 25)	The HCC EV-derived molecular signatures exhibit great potential for noninvasive early detection of HCC from at-risk cirrhotic patients.	EV purification system (Click Chip), fluorescence microscopy, transmission electron microscopy and dynamic light scattering	Sun N, et al. Nature Comm. 2020. [78]
Hepatobiliary Tumors (HCC)	HepPar1+, CD144+, CD162+	HCC patients (n = 15); liver cirrhosis (n = 5); and healthy controls (n = 5)	Levels of HepPar1+ MPs, measured before liver resection, were significantly higher in those who displayed early recurrence compared to those without recurrence. Endothelial-derived EVs (CD144+) or activated endothelial EVs (CD144+/CD62+) were not associated with HCC.	Flow cytometry	Abbate V, et al. Int J Mol Sci. 2017. [79]
Hepatobiliary Tumors (HCC)	miR-122, miR-148a, and miR-1246	HCC patients (n = 5); liver cirrhosis (n = 5)	Serum exosomal level of miR-122, miR-148a, and miR-1246 was significantly higher in HCC than LC and normal control.	Transmission electron microscopy and western blot	Wang Y, et al. Med. 2018. [83]
Hepatobiliary Tumors (HCC)	miR-21	HCC patients (n = 30); CHB patients (n = 30); healthy controls (n = 30)	miR-21 is enriched in serum exosomes which provides increased sensitivity for HCC detection than whole serum.	Transmission electron microscopy and western blot	Wang H, et al. Biomed Res Int. 2014. [79]
Hepatobiliary Tumors (HCC)	GRP78 and Asgr2 miR-10b-5Pp, miR-221-3p, miR-223-3p, miR-21-5p	HCV patients (n = 54); HBV patients (n = 40) HCC patients without HBV/HCV infection (n = 10)	Along with miR-21-5p, miR-10b-5p/miR-221-3p/miR-223-3p was found significantly upregulated in the exosome of HCC.Altered circulating hepatocyte-specific exosomal miRNAs were a risk factor for HCC development in both hepatitis B virus- and hepatitis C virus-infected patients.	NanoSight, transmission electron microscopy, and immune-blotting	Ghosh S, et al. Int J Cancer 2020. [86]
Hepatobiliary Tumors (HCC)	LINC00853	HCC patients (n = 90); chronic hepatitis (n = 28); liver cirrhosis (n = 35); healthy controls (n = 29)	Levels of EV-LINC00853 were higher in HCC patients. EV-LINC00853 displayed excellent discriminatory ability in the diagnosis of all stages of HCC.	ExoQuick	Kim S et al. Mol Oncol. 2020. [87]
Hepatobiliary Tumors (CCA)	-	CCA patients (n = 5); pancreatic cancer (n = 20); nonmalignant (n = 15)	The median concentration of EVs was significantly higher in bile samples from patients with malignant common bile duct stenoses compare to controls or nonmalignant common bile duct stenoses.	NanoSight, transmission electron, and nanoparticle tracking analysis	Severino V et al. Gastroenterology 2017. [90]
Hepatobiliary Tumors (CCA)	CD9, CD63, CD81	CCA patients (n = 43); HCC patients (n = 29); primary sclerosing cholangitis (PSC) (n = 30); and healthy control (n = 32).	Decrease in the EV size in CCA versus PSC patients.HCC patients showed a slight increase in serum EV concentration compared to the other three groups. The selection of biomarkers between CCA vs. control indicated that aminopeptidase N (AMPN), pantetheinase (VNN1), and polymeric immunoglobulin receptor (PIGR) show the best diagnostic capacity.Protein levels of VNN1, C-reactive protein (CRP), FIBG, IGHA1, A1AG1, and gamma-glutamyltransferase 1 are increased in serum EV of CCA patients compared to the other groups.	NanoSight, transmission electron, and nanoparticle tracking analysis	Arbelaiz A, et al. Hepatol. 2017 [91]

## 5. EVs as Therapeutic Tool

EV-based therapeutic approaches have two aims: (a) EVs as therapeutic targets, or (b) EVs as a delivery system of drugs.

In preclinical studies, EVs have been already used as drug delivery carriers mainly for cancer therapy [18]. The main benefit of a drug delivery system is to increase the stability of the drug, increase the therapeutic efficacy, improve the delivery site, and decrease drug resistance [92]. The most known and clinically approved delivery system available are liposomes. Liposome composition, structure, and size are very similar to EVs but the membrane is more complex, improving their site of action. In addition, due to their natural origin, they cannot be recognized by the immune system. Another approach has been to modify proteins at the surface of EVs in order to specify the target site. One approach was to genetically modify cells to promote the expression of specific proteins or RNAs in EVs [93,94]. Ligands can also be introduced in the surface of the EVs by chemical reactions, but this modifies the membrane composition, interfering with the natural function and ability of EVs. 

In order to introduce therapeutic agents, nucleic acids, or proteins into the EVs, several approaches have been described so far, such as cell modification to promote specific EV production or different drug-loading techniques in purified EVs. The methods for drug encapsulation are simple mixing, electroporation, sonication, transfection, and saponin-induced pore formation [18]. As an example, a recent study found that the same dose of EV-encapsulated methotrexate had a greater effect in promoting cell death than the free drug (23% vs 2%) in a cell line of HCC, which confirmed its role as a delivery system [95]. 

It is well known that mesenchymal stem cells (MSCs) are involved in tissue repair mainly by the paracrine release of factors inside EVs [96]. MSC-derived EVs have been shown to activate the regenerative mechanism of the liver, stimulating hepatocyte proliferation and decreasing apoptosis. For instance, the therapeutic effect of MSC and their derived EVs were studied in a lethal murine model of hepatic failure [97]. EV administration reduced hepatic injury and increased survival. A higher concentration of Y-RNA-1 was identified inside the EVs as being responsible for the protective effects of MSC-EVs. The same effect was found by Tan and colleagues. MSC-derived exosomes were administered in a CCl4-induced liver injury mouse model. Exosome treatment attenuated the liver injury, finding a significant increase in hepatic proliferation, which suggests that MSC-derived exosomes can be proposed as a hepatoprotective tool [98]. The potential use of human MSC EVs in attenuating liver damage after hepatic ischemia-reperfusion injury was also studied [99]. Several pieces of evidence suggest the use of stem cell-derived EV therapy for liver regeneration [61] 

The effect of EVs has been also studied in liver fibrosis. Several studies showed that the administration of EVs of different origins alleviates hepatic inflammation and collagen deposition in animals with CCl4-induced liver fibrosis. Povero and colleagues showed that EVs isolated from induced pluripotent stem cells (iPSC) modulate HSC activation, having an antifibrotic effect. HSCs were activated with tumor growth factor, and after that, they were exposed to iPSC-EVs. EV treatment resulted in a decrease of alpha-smooth muscle actin (alpha-SMA), collagen, and fibronectin (profibrogenic markers). Genomic studies revealed that miRNA-92-3p was the most abundant in these iPSC-EVs. Intravenous injection of iPSC-EVs in two animal models of liver fibrosis, CCl4 and bile duct ligation, showed antifibrotic effects at protein and gene levels, and is being proposed as a novel antifibrotic approach [100]. Human bone mesenchymal stem cell-derived exosomes (hBM-MSC-Ex) alleviated liver fibrosis, decreased liver inflammation and collagen deposition, enhanced liver function, and increased hepatocyte regeneration in 8-week CCl4-induced liver fibrosis rats. Significant downregulation of Wnt/β-catenin pathway components was found after hBM-MSC-Ex treatment suggesting that hBM-MSC-Ex could ameliorate liver fibrosis via the inhibition of HSC activation through the Wnt/β-catenin pathway [101]. Recently, another study showed that EVs purified from healthy mice attenuate the profibrogenic activities of HSCs in the CCl4 injury model at 10 days and after 5 weeks. The cargo of these EVs was analyzed showing that significant differences of the 233 CCl4-regulated hepatic gene expression were found mainly associated with fibrosis, cell cycle, cell division, signal transduction, extracellular matrix (ECM), heat shock, cytochromes, drug detoxification, adaptive immunity, and membrane trafficking [102]. Another study modified human umbilical cord perivascular cells (HUCPVCs) in order to overexpress Insulin-like Growth Factor-I (IGF-I). Overexpression was achieved using a specific adenovirus and EVs were isolated. Treatment with EVs enriched in IGF-I significantly reduced the activation of HSCs in vitro and in thioacetamide-induced liver fibrosis mice [103]. 

Circulating EVs are not only increased in human and animal models of NASH [8], but hepatocyte-derived EVs from an in vitro NAFLD system were found to induce the upregulation of fibrogenesis markers (alpha-SMA, collagen, and TIMP-2) in HSCs [104]. In an animal model of NASH, EVs from hepatic liver stem cells reduced liver fibrosis and inflammation, and improved liver function [105]. 

The EV therapeutic approach has been shown to have some advantages compared to cell-based therapy. EVs are more stable, they contain various biological molecules, genetic material, proteins and lipids, and they enter the cells more easily through biological barriers. As EVs come from different types of cells, they can be manipulated in order to express the cargo of interest. However, many challenges need to be overcome in order to prioritize the use of EV therapies in liver disease.

## 6. Conclusions

The field of extracellular vesicles has grown exponentially over the past two decades. Interest in EVs is growing because they are detectable in several fluids, contain a lipid bilayer membrane that protects the encapsulated material, and contain genetic material and proteins from their parent cells that could be transferred to another cell, altering its function, acting as a complex cell-to-cell form of communication mediating diverse biological functions. Several studies have recognized their value as a liquid biopsy biomarker in several acute and chronic diseases, including liver disease. In this review, we provide strong evidence for the use of EVs as a biomarker in several liver diseases, showing high specificity and sensibility for diagnosis and also monitoring the response to treatment in viral or alcoholic hepatitis, NAFLD, and HCC. 

Currently, there is a lack of reproducibility in EV research, which makes it difficult to understand the biology and weakens their potential therapeutic use. Therefore, in the near future, studies need to be directed towards the development of new techniques for isolation and characterization, and also towards establishing standardized protocols for processing samples. Improving the reproducibility will make it possible to extrapolate findings between different EV studies, and finally enhance their clinical utility.

## Data Availability

Not applicable.
